# S136. A NOVEL APPROACH FOR DEVELOPING PREDICTION MODEL OF TRANSITION TO PSYCHOSIS: DYNAMIC PREDICTION USING JOINT MODELLING

**DOI:** 10.1093/schbul/sby018.923

**Published:** 2018-04-01

**Authors:** Hok Pan Yuen, Andrew Mackinnon, Jessica Hartmann, Paul Amminger, Connie Markulev, Suzie Lavoie, Miriam Schafer, Andrea Polari, Nilufar Mossaheb, Monika Schlogelhofer, Stefan Smesny, Ian Hickie, Gregor Berger, Eric Chen, Lieuwe de Hann, Dorien Nieman, Merete Nordentoft, Anita Riecher-Rössler, Swapna Verma, Andrew Thompson, Alison Yung, Patrick McGorry, Barnaby Nelson

**Affiliations:** 1 Orygen, The National Centre of Excellence in Youth Mental Health, Centre for Youth Mental Health, The University of Melbourne; 2 Centre for Mental Health, The University of Melbourne, Black Dog Institute and University of New South Wales; 3 Orygen, the National Centre of Excellence in Youth Mental Health; 4 Medical University of Vienna; 5 University Hospital Jena; 6 Brain & Mind Research Institute, University of Sydney; 7 Child and Adolescent Psychiatric Service of the Canton of Zurich; 8 University of Hong Kong; 9 Academic Medical Center, Amsterdam; 10 Mental Health Centre Copenhagen, Mental Health Services in the Capital Region, Copenhagen University Hospital; 11 Psychiatric University Clinics Basel; 12 Institute of Mental Health, Singapore; 13 Warwick Medical School, University of Warwick, North Warwickshire Early Intervention in Psychosis Service, Coventry and Warwickshire NHS Partnership Trust; 14 Institute of Brain, Behaviour and Mental Health, University of Manchester, Greater Manchester West NHS Mental Health Foundation Trust

## Abstract

**Background:**

Ever since the establishment of strategies for identifying people at ultra-high risk (UHR) of developing psychosis about twenty years ago, much research has been conducted in seeking risk factors and in developing prediction models for predicting which UHR individuals will actually make a transition to psychosis. The goal is to provide specific interventions to those of high susceptibility. Such research almost invariably uses fixed predictor variables, typically variables assessed at baseline, i.e. service entry. Interest has now emerged to investigate whether the dynamic nature of psychopathology can be used to improve prediction of the onset of psychosis. As studies on UHR individuals usually require follow-up of participants over time, the longitudinal nature of these studies provides the opportunity to capture the dynamic characteristics of psychopathology by conducting multiple assessments across the study period. The idea is that prediction can be updated continuously as more information about changes in patients’ conditions are obtained. Over the past two decades, statistical methodology that can combine the time-to-transition aspect and the longitudinal aspect of UHR studies into one model has emerged. The methodology is called joint modelling.

**Methods:**

The aim is to describe the joint modelling methodology and to demonstrate how joint modelling can be used to develop a prediction model for transition to psychosis. The data from the NEURAPRO Study was used for the demonstration. This study was a multi-centre placebo-controlled randomized trial of the effect of omega-3 polyunsaturated fatty acids on transition risk in UHR individuals. The sample size was 304. Study assessments were conducted monthly during the first 6 months and then at months 9 and 12. There were in total 40 known transitions.

**Results:**

Compared with the conventional approach of using only fixed predictors, joint modelling prediction models showed significantly better sensitivity, specificity and likelihood ratios.

**Discussion:**

Joint modelling is a useful statistical tool which can improve the prediction of the onset of psychosis and has the potential in guiding the provision of timely and personalized treatment to patients concerned.

